# Azoospermia in rabbits following an intravas injection of Vasalgel ™

**DOI:** 10.1186/s12610-016-0033-8

**Published:** 2016-03-30

**Authors:** Donald Waller, David Bolick, Elaine Lissner, Christopher Premanandan, Gary Gamerman

**Affiliations:** Prelabs, LLC Inc., 33 W Chicago Ave., Oak Park, IL 60302 USA; Seraphim Life Sciences Consulting, LLC, 2158 Bonaventure Drive, Suite 101, Vienna, VA 22181 USA; Parsemus Foundation, PO Box 2246, Berkeley, CA 94702 USA; Department of Veterinary Biosciences, College of Veterinary Medicine, The Ohio State University, 1925 Coffey Road, Columbus, OH 43215 USA

**Keywords:** Male contraception, Hydrogel, Vas deferens, Styrene maleic acid, Polymer, Vasectomy

## Abstract

**Background:**

Vasectomy is currently the only long-acting contraceptive option available for men, despite increasing demand and potentially significant positive impacts on human health of additional male contraceptive options. Vasalgel ™ is a high molecular weight hydrogel polymer being developed as a non-hormonal long-acting reversible male contraceptive. Vasalgel consists of styrene-alt-maleic acid dissolved in dimethyl sulfoxide, which is distinct from styrene-alt-maleic anhydride materials previously studied.

**Methods:**

The goal of the study was to determine the contraceptive efficacy of two test articles with different levels of styrene maleic acid (100 %, and 80 % acid/20 % anhydride). The test articles were injected bilaterally in the vasa deferentia of mature male rabbits. Post-implantation analyses of semen parameters were completed over a 12 month period and compared to baseline measures of sperm concentration, motility and forward progression.

**Results:**

Both test articles were effective in blocking the passage of spermatozoa through the vasa deferentia in the 12 subjects completing the study. A significant decrease in sperm concentration occurred following implantation of the test material, with no measurable sperm concentration except for a few samples in one animal that were markedly oligospermic. Vasalgel produced a rapid onset of azoospermia, with no sperm in semen samples collected as early as 29–36 days post-implantation, and was durable over a 12 month period.

**Conclusion:**

This study indicated that Vasalgel is an effective non-hormonal long-acting male contraceptive in a rabbit model.

## Background

The availability and use of contraceptives has far-reaching implications for human health and wellbeing. An estimated 85 million unintended pregnancies occur annually worldwide, with half ending in abortion and 13 % in miscarriage [1]. The unmet need for modern contraception in developing countries was estimated at 222 million in 2012. Serving this need would prevent 54 million unintended pregnancies, including 26 million abortions [2]. Even when available, male and female contraceptive methods are often used inconsistently or discontinued early for a myriad of reasons (e.g., lack of access to product supply or prescriptions; unwanted side effects or health concerns; seeking a more effective method), accounting for 33 million of the accidental pregnancies worldwide [3, 4].

While several options for long-acting contraception are available to women, vasectomy is the sole long-acting method available to men. Vasectomy is safe and very effective but is generally considered permanent because its reversal is technically difficult, expensive, and may not be successful in restoring fertility (e.g., [5]). Reversible male methods of contraception (male condom, spermicide, periodic abstinence and withdrawal) have relatively high failure rates and are not well accepted by various populations [6]. A large number of potential male contraceptives have been studied, including hormonal methods with ongoing clinical trials [7]. However, given the risks and known side effects for female hormonal birth control, ranging from venous thromboembolism to excessive menstrual bleeding, risks may be anticipated in a hormonal approach for men as well, and focusing effort on non-hormonal alternatives is important [8]. Demand for a non-hormonal, highly effective contraceptive for men is increasing, with uptake expected to reduce the incidence of unplanned pregnancy and its related sequelae [9].

A likely target for non-hormonal male contraception is the vas deferens, which carries sperm from the epididymis to the ejaculatory duct. A number of products have been tested that do not allow viable sperm to pass through the vasa deferentia [10]. Examples include solid intra-vas occlusion devices (made of polyurethane or silicone), vas clips, and copper mesh filtering devices [11–15]. Most vas-occlusive products have suffered from limited reversibility, side effects or problems with long term efficacy.

Intra-vas injections of polymers that become hydrogels are very promising as a device for male contraception. One product, called RISUG® (Reversible Inhibition of Sperm Under Guidance) based on styrene maleic anhydride (SMA anhydride), has been studied in India for over three decades [16]. The researchers report long-term efficacy in preclinical and human studies [17, 18], with Phase III clinical trials underway since 2002. Fertility was returned in rats following removal of the material by flushing with bicarbonate [19, 20] and in monkeys through palpation and percutaneous electrical and vibratory stimulation [21–23]. RISUG reportedly works by blocking sperm transport, creating an incompatible pH level, and generating a positive charge that ruptures the acrosomal membrane of the negatively charged sperm head [10].

Vasalgel™ is a high molecular weight polymer being developed in the US as a contraceptive device for men. While both entail an injection into the vas deferens, Vasalgel has important differences in composition and function when compared to RISUG. Vasalgel is comprised mainly of styrene-alt-maleic acid (SMA acid). Because SMA anhydrides may hydrolyze in aqueous conditions, SMA acid without SMA anhydride has the potential advantages of a less complex process of production and long term stability without concern for hydrolysis, creating a more feasible regulatory path. After implantation, the SMA acid forms a hydrogel that appears to be tissue adherent, fills the lumen of the vas deferens and acts as a mechanical barrier to the passage of sperm. The purpose of the present study was to determine the efficacy of the Vasalgel SMA polymer hydrogel to produce rapid and durable contraception over 12 months in a rabbit model. Because efficacy of primarily SMA anhydride preparations (e.g. RISUG) is known and efficacy of SMA acid preparations was neither known nor assured, preparations of SMA acid and mixed SMA acid and anhydride were compared.

## Methods

### Test articles

Vasalgel test articles consisted of 25 % solutions by weight of SMA in DMSO. The average molecular weight (Mw) of the SMA anhydride (Poly(Styrene-co-Maleic Anhydride, CAS Registry Number: 9011-13-6) was 330 kDa according to standardized gel permeation chromatography (GPC) methodology (Jordi Labs, Mansfield, MA, USA). The SMA acid (Poly(styrene-*alt*-maleic acid, CAS Registry Number: 25736-61-2**)** was made by hydrolysis of the anhydride and had a Mw of 360 kDa. Test article #1 contained only SMA acid (referred to as “100 % acid”). Test article #2 was prepared by weighing appropriate amounts of the SMA acid and SMA Anhydride and to achieve a mixture of 80 % SMA acid and 20 % SMA anhydride by weight (referred to as “80:20 mix”) in DMSO. The final test articles were prepared and packaged in a nitrogen atmosphere in 2 ml glass vials by Polysciences, Inc. (Warrington, PA, USA). The 80:20 mix was selected to provide a comparison with the 100 % SMA Acid. This was based upon an evaluation of the material responses in the presence in saline. An increase in the amount of anhydride in the mix resulted in a less flexible and harder material which was not desired within the vas.

### Subjects, housing and care

The studies were performed using 15 mature male and three mature female New Zealand White rabbits that averaged 26.2 weeks of age (SD = 0.37 weeks) and weighed an average of 3.7 kg (SD = 0.10 kg) on arrival from Harlan Laboratories, Oxford, MI. The animals were screened for health conditions and acclimated for 7 days. Housing and care protocols used recommendations of the National Centre for the Replacement, Refinement, and Reduction of Animals in Research [24]. Rabbits were individually housed in stainless steel cages in temperature- and light- controlled rooms meeting the requirements specified in the FELASA publication entitled Euroguide [25] and fed Purina® Rabbit Chow™ (Purina Mills, St. Louis, MO, USA). The rabbits also had regular access to a play area within the same room, where they could socialize and exercise. All animals were checked at least twice daily for viability and general health during the entire period of the study. The female rabbits were only used as teaser animals when collecting semen specimens. They were housed in the same room as the male rabbits and were placed for adoption at the end of the study. All animal procedures were approved by the Loyola University Chicago Institutional Animal Care and Use Committee.

### Experimental design

Baseline semen sample collection was attempted for all 15 male rabbits for an average of 6 weeks (SD = 2.7 weeks) prior to implantation of the Vasalgel device. All rabbits then received bilateral vas deferens implants of 100 % acid (*n* = 8) or 80:20 mix (*n* = 7). Semen collection began again at an average of 10.8 weeks (SD = 4.9 weeks) post implantation. Semen collection continued approximately weekly until azoospermia was observed. Semen was then collected twice monthly until approximately 12 months post injection.

### Implantation of test articles

Animals were weighed, given an antibiotic (Baytril® [Bayer Healthcare, KS, USA] 5 mg/kg) and then anesthetized with an intramuscular injection of xylazine HCl (4 mg/kg) and ketamine HCl (50 mg/kg) and a subcutaneous injection of acepromazine maleate (1.0 mg/kg). A 1 cm suprapubic transverse incision was made in the midline approximately 2 cm cephalad to the pubic symphysis. The spermatic cords were brought up through the incision and isolated. The cremasteric fascia was incised in a longitudinal fashion and the vas deferens isolated with its blood supply.

The isolated right and left vasa deferentia were elevated and injected with approximately 100–120 μl of test article in about 30–40 seconds using a 24 gauge 1.6 cm catheter (Quik-Cath by Baxter, Deerfield, IL). This volume of material was expected to fill the vas deferens to an approximate length of 2 cm based on preliminary studies in euthanized rabbits. The catheter was then removed, the vasa deferentia gently compressed for about 30 s and the vasal muscularis at the site of injection identified with a 6-0 Prolene suture. The vas deferens was returned to the spermatic cord and the site closed with 4-0 nylon sutures. The rabbits were given antibiotic Baytril® (5 mg/kg) (Bayer Healthcare, KS, USA) once per day for 7 days post-operatively and pain medication buprenorphine HCl (0.2 ml) every eight hours for 72 h post-operatively. All surgical procedures were performed by the same surgical team.

### Semen collection and evaluation

Semen collections were performed using a warmed artificial vagina semen-collection device designed for use with the rabbit and a “teaser” female to encourage mounting (e.g., [26]). Semen specimens were evaluated for volume, total sperm count, sperm motility and forward progression using standard manual methods. Additionally, any observations of possible sperm were recorded when there were too few sperm to count using standard methods.

### Euthanasia and necropsy

Rabbits that died during the 23 month study received a necropsy following standard protocol with particular attention to the reproductive tract.

The vas deferens from six animals were harvested and immersed in 10 % neutral buffered formalin for fixation. The vasa were from one animal that died three days after implant, three animals necropsied during the course of the study due to not providing semen samples or behavioral issues (Days 189, 214 and 248) and two after extended exposure to Vasalgel (Days 572 and 538). The tissues were processed, sectioned and stained with hematoxylin and eosin utilizing standard methods for evaluation.

### Data analysis

Data were summarized by subject. The efficacy of the test article to block sperm was evaluated by comparing the mean sperm parameters (sperm concentration, forward progression and motility) for each subject by condition (baseline vs. post-implant) and by test article (100 % acid, 80:20 mix). Repeated measures analysis of variance using a 2 × 2 design was used to statistically test the condition x test article significance using the program Statistica (StatSoft, Inc. Tulsa, OK, USA). *T*-test for independent samples was conducted to determine any difference in sperm parameters at baseline. A significance level of *p* < 0.05 was determined. Data are presented as mean ± standard deviation.

## Results

Injection of the test material was successfully accomplished in all male subjects. The time to complete the implantation surgery decreased 33 % from the first five to the last five procedures (54.0 ± 21.6 vs. 36.0 ± 10.8 min) as the surgeon gained experience identifying and injecting the small and fragile rabbit vasa deferentia. Extravasation of a small amount of test material occurred in four vasa deferentia and one became distended when the material was injected.

Twelve animals (*n* = 6 in each of the 100 % acid and 80:20 mix groups) from the original 15 were retained in the study. Three rabbits died postoperatively without precipitating illness, apparently from surgical trauma as the operative technique was being refined. In two of the subjects (one 100 % acid, one 80:20 mix), the reproductive tract became engorged with blood caudal to the injection site, involving a single vas deferens in one male and both testes, vasa deferentia and epididymides in the other male. There were no direct observations of inflammation due to the presence of Vasalgel within the vasa. All other organs appeared normal. The third male (100 % acid) was found dead three days after surgery with no obvious cause. All organs, including the reproductive tract, appeared normal and the incision site was healing normally. There were no direct observations of any inflammation or tissue damage due to the presence of Vasalgel. One of the twelve study subjects died six months following the implant surgery due to neck trauma unrelated to surgery or the presence of Vasalgel. The reproductive tract appeared normal and his data were included in analyses. See Table 1 for information on each subject.

**Table 1:** Number of semen samples and sperm concentration during baseline and after implantation of the test articles for each subject

		Baseline		Implantation		
Animal ID	Test article	# Semen samples	Sperm Concentration × 10^6^ sperm/ml (mean ± SD)	# Semen samples	Sperm Concentration × 10^6^ sperm/ml (mean ± SD)	Outcome
8949	100 % acid	3	228.3 ± 78.5		0.00	
8960	100 % acid	3	211.7 ± 175.3	4	0.00	Died six months after implantation
8963	100 % acid	3	194.3 ± 146.3	13	0.00	
8964	100 % acid	2	414.0 ± 179.6	24	0.00	
8968	100 % acid	2	165.5 ± 170.4	22	0.00	
8972	100 % acid	3	189.0 ± 52.5	24	0.00	
8956	80:20 mix	3	130.7 ± 76.3	18	0.00	
8958	80:20 mix	3	148.0 ± 59.3	27	0.00	
8965	80:20 mix	2	296.5 ± 146.4	28	0.24 ± 1.0	
8966	80:20 mix	2	324.0 ± 107.5	23	0.00	
8970	80:20 mix	3	267.3 ± 170.0	8	0.00	
8971	80:20 mix	2	228.0 ± 63.6	25	0.00	

# = number

A total of 264 semen samples were successfully collected (of 459 attempts) from the 12 subjects over the course of the study (Table 1). Most unsuccessful attempts were due to ejaculation outside the artificial vagina. An average of 2.6 ± 0.5 samples were collected per subject in the baseline condition and 19.4 ± 7.6 were collected per subject over the 12 months following implantation. Semen volume was 0.30 ± 0.07 ml during baseline and 0.76 ± 0.30 ml after implantation of the test article.

Significant decreases in sperm measurements occurred in all 12 animals following implant of the test article (Table 1). Sperm concentration during baseline was 233.1 ± 81.2 × 10^6^ sperm/ml (range of individual averages 130.7 – 324.0 × 10^6^ sperm/ml). Semen samples following implantation contained no measurable sperm using standard methods during the 12 month follow-up period, except for one rabbit with very low sperm concentrations measured in five of the first 13 semen samples but eventually provided semen samples with no spermatozoa present in the ejaculate. The average post-implantation sperm concentration from this animal was 0.24 × 10^6^ sperm/ml.

At baseline, the mean percentage of motile sperm was 78.2 ± 6.8 and the mean percentage showing forward progression was 42.5 ± 7.2. Following implant, these parameters were zero in all samples.

Semen analysis also included qualitative observations of small numbers of sperm or fragments in the samples, which were too few to reliably count using standard methods. Of 233 samples collected after implantation with the contraceptive, three rabbits in the 80:20 mix group had very few sperm in their first semen sample (occurring at day 29, 35 and 64 post-implant) indicating that virtually all sperm were blocked.

**Fig. 1:**
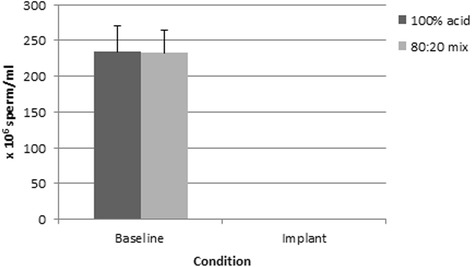
Sperm concentration during baseline and implant conditions for the two test articles. Difference between conditions was significant (*p* < 0.001)

There was no significant difference in the three baseline semen parameters for the two test articles (all *p* > 0.2). Statistical analysis of the impact of condition (baseline vs. implant) by test article (100 % vs 80:20 mix) indicated a significant condition effect with sperm concentration being significantly lower during the implant condition (*F* = 90.0, *p* < 0.001) (Fig. 1). The test article was not influential in this relationship since the test article and condition x test article interactions were not significant (*p* > 0.05). It was not possible to conduct a similar statistical test on the motility and forward progression because there was no variability in the implant condition (due to zero motility and progression values).

**Fig. 2:**
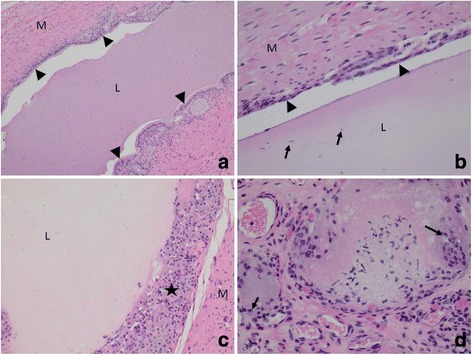
Rabbit ductus deferens containing SMA hydrogel. **a** Longitudinal section (100X magnification) of vas deferens containing hydrogel appearing as eosinophilic homogenous luminal material. Arrowheads depict the mucosal epithelium. Muscularis (M), vas deferens lumen (L). **b** Longitudinal section (400X magnification) of vas deferens containing hydrogel. Arrowheads depict the attenuated mucosal epithelium in this section. Scattered spermatozoa are present (arrows). Muscularis (M), vas deferens lumen (L). **c** Cross section (200X magnification) of vas deferens. A layer of epithelioid macrophages replaces the luminal epithelium in this section (★). Muscularis (M), vas deferens lumen (L). **d** Interstitial hydrogel with associated granulomatous inflammation (400X magnification). Arrows depict epithelioid macrophages and multinucleated giant cells surrounding eosinophilic to amphophilic material in the interstitium

Histological evaluation of the vasa deferentia of six subjects was completed. The vas deferens in the one animal after three days of implant exposure exhibited an intraluminal accumulation of non-fibrillar eosinophilic to amphophilic homogenous material (Fig. 2a). The material accumulated in the lumen was devoid of inflammatory cells and epithelial cells. One animal exhibited a moderate accumulation of spermatozoa embedded in the material and the remaining animals evaluated exhibited scattered spermatozoa or no spermatozoa embedded in the intraluminal material. The mucosal epithelium in all animals demonstrated some degree of attenuation, taking on a cuboidal to flattened appearance in contrast to the ciliated columnar epithelium observed in regions devoid of intraluminal material. Segmental mucosal epithelial loss without inflammation was observed. In some treated animals, the mucosal epithelium was replaced by round to polygonal cells with abundant eosinophilic cytoplasm and round to oval nuclei (Fig. 2b,c). These cells often exhibited close association to each other and were interpreted as epithelioid macrophages. Larger cells were also present with similar cytoplasm but contained multiple round to oval nuclei (ranging from 2–10 nuclei within a cell), interpreted as multinucleated giant cells. This replacement of mucosal epithelium was observed in both a segmental and a circumferential manner in the treated animals.

Similar material present in the lumen of the vas deferens was also observed in the some surrounding interstitial connective tissue interpreted as extraluminal hydrogel (Fig. 2d). The accumulation of this material was often arranged in a multifocal to coalescing nodular pattern and was associated with a surrounding rim of epithelioid macrophages and multinucleated giant cells. The multinucleated giant cells observed in the interstitial space frequently exhibited higher nuclei numbers than those seen at the mucosal surface of the vas deferens. Both test articles (100 % SMA and 80:20 mix) were similar in the degree of epithelial attenuation present as well as the inflammation present at the mucosa and in the interstitium.

The histological observations did not appear to be dependent upon the duration of Vasalgel exposure.

## Discussion

Despite the need for male contraception and the fact that vas-occlusive devices have been tested for decades, no product has been successfully introduced to the market. The characteristics of the occlusive material are critical and previous attempts to use solid or semi-solid material had side effects or unacceptable efficacy. For example, a urethane intra-vas device tested in 288 men in China proved to be only 94.3 % effective, not matching vasectomy’s 98.6 % effectiveness in the study [12].

The characteristics of the Vasalgel test articles likely influenced the ability of the hydrogels to provide effective contraception [16]. Previous investigators of styrene-based vas deferens contraceptives have reported efficacy with anhydride-based formulations [16]. However, anhydrides are susceptible to hydrolysis to an acid over time in the presence of water. A 100 % SMA acid-based formulation, rather than a styrene maleic anhydride formulation should result in greater stability under long term storage conditions. This study addresses the question of whether a 100 % acid SMA formulation will provide the same efficacy as an anhydride or acid-anhydride mix formulation. The current study demonstrate that both 100 % acid and 80:20 mix formulas dissolved in DMSO were effective in blocking the vas deferens to the passage of spermatozoa. A significant decrease in sperm concentration occurred following implantation of the test material, with 99.6 % of semen samples collected having no measurable sperm concentration. Although a non-significant finding, the efficacy of the 100 % acid test article was supported by the finding that the only rabbits that had small numbers of sperm in the initial semen samples were from the 80:20 mix group.

The onset of azoospermia was rapid and durable over the 12 month period. Rabbits that had samples collected as early as 29–36 days post implantation were azoospermic. As described above, qualitative observations indicated that only three subjects evidenced a few sperm visible in their first sample. Histologically, vas deferens from both the 100 % SMA group and the 80:20 mix group exhibited mucosal flattening with epithelial attenuation. Granulomatous inflammation was seen in some animals in both groups and manifested as either mucosal replacement by the inflammatory cell population or interstitial nodular inflammation with intralesional polymer. The presence of some extraluminal gel may have occurred by extravasation of the material immediately following injection. However, the tissue response appears to be minimal with characteristics of a normal foreign body response. These changed appear to be similar in the vasa from both the short term and the longer term exposures to Vasalgel.

The SMA acid polymer dissolved in DMSO appears to provide functional advantages as a contraceptive. Advantages may stem from the ability of DMSO to rapidly transit through an aqueous milieu as well as cells and tissues [27], resulting in diffusion of DMSO from the vasa deferentia and formation of a flexible, tissue adherent hydrogel. The maleic acid groups in SMA are thought to be essential to hydrogel formation and hydrolysis of the maleic anhydride groups in the 80:20 mix implants to the maleic acid form of the SMA may also facilitate hydrogel formation [28]. Hydrogels allow transit of many water soluble molecules but not larger structures such as spermatozoa, and this hydropermeability may reduce hydrostatic pressure in the epididymis and rete testis. The implant remains in a soft gel-like state, with the ability to flex and adhere to the vasa deferentia and minimize any accommodation of the vas to the presence of the test article. This is in contrast to solid vas plug devices, which may have failed due to an increase of the vas diameter from the presence of a solid material within the lumen of the vasa. A dilation of the vas has been observed in past studies when a solid plug was inserted into a vas for an extended period of time which can then allow passage of the spermatozoa around the solid plug [Waller D (2012) Unpublished observations].

Three rabbits died following the surgery from apparent surgical trauma. One was implanted with 80:20 mix and two were implanted with the 100 % acid formulations. None of the deaths appeared to be related to the presence of the injected material. The anatomy of the rabbit dictates abdominal surgery to access and inject the vasa deferentia, which entails higher surgical risk and trauma than accessing the human vas. In humans, the Vasalgel contraceptive implantation can be conducted using the minimally invasive No Scalpel Vasectomy procedure to access and inject Vasalgel [29].

It has been suggested that the method of contraceptive action of the primarily anhydride SMA material (RISUG) was through deactivating sperm passing by the gel, which lined the walls of the vas lumen without complete occlusion. This was reportedly achieved because the hydrogel lowered pH and induced electrical charge disturbances that impaired acrosome function [16, 30, 31]. In this study, we found no evidence that measurable numbers of sperm passed through the primarily SMA acid material. The Vasalgel product appears to be effective by blocking sperm from passing through the vas deferens as its primary mode of action.

## Conclusions

Both 100 % SMA acid and the 80:20 mix test articles injected into the rabbit vasa deferentia produced rapid onset of azoospermia that was durable throughout a 12 month period, proving effectiveness in the rabbit model. The effects on the structure of the vas appear to be minor and support the potential for a return to full function following removal. Further study of this new contraceptive, including success in flushing the gel from the vasa deferentia to return sperm flow, is needed to confirm its potential as a long-acting reversible male contraceptive.

## Abbreviations

GPC: gel permeation chromatography
Mw: molecular weight
SD: standard deviation
SMA acid: styrene maleic acid
SMA anhydride: styrene maleic anhydride
